# THE LONG-TERM RELATIONSHIP BETWEEN BMI AND COGNITIVE PERFORMANCE: A CROSS-LAGGED PANEL ANALYSIS

**DOI:** 10.1093/geroni/igad104.2138

**Published:** 2023-12-21

**Authors:** Andrew Fiscella, Ross Andel

**Affiliations:** University of South Florida, Tampa, Florida, United States; Arizona State University, Tempe, Arizona, United States

## Abstract

While obesity has traditionally been associated with negative outcomes, an obesity paradox has been observed which suggests that older adults may show some benefit from having a higher weight. This longitudinal study examined the association between body mass index (BMI) and episodic memory performance. Data from 14,639 participants collected as part of the Health and Retirement Study were used in a 10-year random intercept cross-lagged panel analysis. BMI was used to measure obesity while T-scores from tests of immediate and delayed recall were used to represent episodic memory performance. Initially, a higher BMI was associated with lower episodic memory scores in the following wave (β= -.132, p<.001), but this association became positive over time (β=.142, p< .001) with higher BMI related to better episodic memory scores, even after accounting for demographic and health covariates. The association between BMI and episodic memory over time was stronger in older adults (β=.068, p<.05) than middle-aged adults (β=.020, p=.533). Additionally, while episodic memory scores were not significantly associated with BMI at the next wave for middle-aged adults (p=.283), they were for older adults (p<.05). These findings provide support for the obesity paradox and are consistent with previous studies employing more traditional longitudinal designs. The results of this study raise questions as to what is behind the association between episodic memory and BMI and why it appears to trend positively in older adulthood. Further studies are needed to examine potential causal mechanisms.

